# Supplementary material for: Securin Regulates the Spatiotemporal Dynamics of Separase
Source: bioRxiv. 2023 Dec 19:2023.12.12.571338. Preprint. [Version 2] doi: 10.1101/2023.12.12.571338 (PMC10760073; doi:10.1101/2023.12.12.571338)
Supplement: Supplement 1 [file NIHPP2023.12.12.571338V2-supplement-1.pdf]

bioRxiv preprint doi: https://doi.org/10.1101/2023.12.12.571338; this version posted December 19, 2023. The copyright holder for this preprint (which was not certified by peer review) is the author/funder, who has granted bioRxiv a license to display the preprint in perpetuity. It is made available under aCC-BY 4.0 International license.

Figure S1. Dynamics of Separase and Securin during meiosis I. (**A-D**) Representative images of meiosis I in embryos co-expressing endogenously tagged SEP-1::mScarlet (red) and IFY-1::GFP (green). (A) At prophase I, IFY-1::GFP is present in both the nucleus (caret) and cytoplasm. SEP-1::mScarlet is cytoplasmic and excluded from the nucleus. (B) At NEBD, SEP-1::mScarlet enters the nucleus and colocalizes with IFY-1::GFP at kinetochores (caret). (C) In prometaphase I, SEP-1::mScarlet and IFY-1::GFP remain co-localized on kinetochore cups (caret), and linear elements (arrow) in the cortex. (D) During anaphase I, SEP-1 localizes to its sites of action at the central spindle (caret) and cortical granules (arrowhead) while IFY-1::GFP is mostly degraded. Scalebar: 10µm. (E) SEP-1::mScarlet (red) and IFY-1::GFP (green) colocalize on the spindle at the metaphase to anaphase transition. Securin is rapidly degraded while separase relocalizes to the midbivalent (caret) at anaphase onset (time shown in seconds, t = 0 is anaphase onset as defined by midbivalent accumulation) and remains highly enriched at the central spindle. (F) Cortical images of embryos expressing SEP-1::mScarlet (red) and IFY-1::GFP (green). SEP-1::mScarlet colocalizes with IFY-1::GFP on linear elements initially (arrows), but securin is largely degraded before separase relocalizes to vesicles (time shown in seconds, t = 0 marks prominent vesicle localization in mid anaphase). (G) Quantification of cytoplasmic securin levels in multiple WT securin GFP lines, compared with GFP::IFY-1^{DM}. Regardless of expression levels, securin is rapidly degraded beginning prior to anaphase onset (t = 0 denotes chromosome segregation in WT and midbivalent localization in mutant). Scale bars: 5µm.

Figure S2. SEP-1 and IFY-1 persist on kinetochore structures after APC/C RNAi. (**A**) Representative image of embryos co-expressing endogenously tagged SEP-1::mScarlet (red) and endogenously tagged IFY-1::GFP (green) after *apc-2* RNAi. The image shown is a cortical slice from a z-series chosen to emphasize SEP-1 and IFY-1 persisting together on kinetochore filaments in the cortex (white arrows).

bioRxiv preprint doi: https://doi.org/10.1101/2023.12.12.571338; this version posted December 19, 2023. The copyright holder for this preprint (which was not certified by peer review) is the author/funder, who has granted bioRxiv a license to display the preprint in perpetuity. It is made available under aCC-BY 4.0 International license.

Numbers correspond to relative position of oocytes and embryos in the hermaphrodite germline. Scale bar: 10µm.

Figure S3. Meiosis I Cohesin dynamics and regulation during meiosis I. Representative images (A-D) of COH-3::GFP (green) with chromosome marker H2B::mCherry (red). (A) Kymograph from an *ex utero* time series at the metaphase-to-anaphase I transition. COH-3::GFP localizes to the midbivalent (caret) until shortly before anaphase I onset (t=0, time indicated in seconds). Representative images of the proximal germline after (B) control, (C) *apc-2* RNAi, and (D) *ify-1* RNAi. (B) In control, COH-3::GFP midbivalent signal is similar in oocytes (-1) and fertilized prometaphase I embryos in the spermatheca (labeled 0) (white arrows), but is absent from embryos after meiosis I in the uterus (+1, +2). (C) After *apc-2* RNAi, COH-3::GFP levels are retained at the midbivalent in prometaphase I embryos (labeled 0) and several arrested embryos in the uterus (+1, +2). (D) After *ify-1* RNAi, COH-3::GFP midbivalent signal prematurely reduced in prometaphase I embryos (labeled 0) relative to oocytes (-1) and is not observed in older embryos (+1). (E) Quantification of COH-3::GFP signal at the midbivalent in different conditions. The ratio between the COH-3::GFP signal on chromosomes in the prometaphase I embryo relative to the -1 oocyte in the same plane was calculated. The COH-3::GFP signal ratio was near 1 in control and *apc-2* RNAi (N=5, $p>0.05$) but was significantly reduced (about 50% reduction) after *ify-1* RNAi (N=4, $p<0.05$). Scale bars: 5µm.

bioRxiv preprint doi: https://doi.org/10.1101/2023.12.12.571338; this version posted December 19, 2023. The copyright holder for this preprint (which was not certified by peer review) is the author/funder, who has granted bioRxiv a license to display the preprint in perpetuity. It is made available under aCC-BY 4.0 International license.

Video Legends

Video 1. **Chromosome dynamics at the metaphase-to-anaphase transition of meiosis I**. Single plane time lapse image series of embryos expressing H2B::mCherry with COH-3::GFP, SEP-1::GFP, GFP::IFY-1^{WT}, or GFP::IFY-1^{DM}. Time (seconds) is normalized to anaphase I onset (t = 0). Playback speed is 7 frames per second.

Video 2. **Separase dynamics at anaphase onset of meiosis I**. Single plane time lapse image series of embryos expressing SEP-1 (shown in green) with H2B, COH-3, or IFY-1^{WT} (shown in red). Time (seconds) is normalized to anaphase I onset (t = 0) as defined by SEP-1 midbivalent enrichment. Playback speed is 7 frames per second.

Video 3. **Securin and separase dynamics in the cortex during anaphase I**. Cortical time series from worms expressing SEP-1 (shown in green) with IFY-1 or RAB-11 (shown in red). Time (seconds) is normalized to anaphase I onset (t = 0), which occurs approximately 30 seconds before SEP-1 disappears from completely from kinetochore filaments. The SEP-1 + IFY-1 movie is a max projection acquired with a 60x objective, while SEP-1 + RAB-11 is a single plane movie acquired with a 100x objective. Playback speed is 7 frames per second.

Video 4. **GFP::IFY-1^{DM} blocks chromosome segregation during meiosis I**. Single plane time lapse image series of embryos expressing H2B::mCherry with GFP::IFY-1^{WT} or GFP::IFY-1^{DM}. Time (seconds) is normalized to GFP enrichment at the midbivalent. Playback speed is 7 frames per second.

Video 5. **Separase localizes to the midbivalent and spindle when GFP::IFY-1^{DM} is overexpressed in anaphase I**. Single plane time lapse image series of embryos expressing SEP-1 (shown in green) with GFP::IFY-1^{WT} or GFP::IFY-1^{DM} (shown in red). Time is shown in seconds, t = 0 occurs when SEP-1 appears at the midbivalent. Playback speed is 5 frames per second.

Video 6. **GFP::IFY-1^{DM} prevents SEP-1 localization to cortical granules during anaphase I**. Cortical max projections from a time lapse image series of embryos expressing SEP-1 (green) and CAV-1 (red) with GFP::IFY-1^{WT} or GFP::IFY-1^{DM} (red). Time (seconds) is normalized to anaphase I onset (t = 0). Playback speed is 3 frames per second.

bioRxiv preprint doi: https://doi.org/10.1101/2023.12.12.571338; this version posted December 19, 2023. The copyright holder for this preprint (which was not certified by peer review) is the author/funder, who has granted bioRxiv a license to display the preprint in perpetuity. It is made available under aCC-BY 4.0 International license.

Table S1. List of *C. elegans* strains used in this study.

Strain	Genotype
DP38	*unc-119(ed3) III*
JAB20	*ltIs151 [pSO33; Pcpg-2::cpg-1SigSeq::mCherry-TEV-STag::cpg-2; unc-119(+)]; unc-119(ed3) III, ojIs58[SEP-1::GFP unc119(+)]; ojIs37[[Ppie-1::H2B::mCherry; unc-119(+)]]*
JAB30	*pie-1p::GFP::ify-1(dm) (pjk3); unc-119(ed3)III*
JAB157	*ojEx75(ify-1::gfp); Itls37[pAA64: pie-1p::mCherry::his-59 + unc119 (+)]*
JAB161	*unc-119(ed3) III; ddIs128[ify-1::TY1::EGFP::3xFLAG(92C12) + unc-119(+)]; ltIs37 [pAA64: pie-1p::mCHERRY::his-58 + unc-119 (+)]*
JAB180	*pie-1p::GFP::ify-1(dm) (pjk3); unc-119(ed3) III*
JAB189	*sep-1(erb-75[sep-1::linker::mCherry])*
JAB195	*sep-1(erb-75[sep-1::linker::mCherry]); unc-119(ed3) III, ojEx75[IFY-1::GFP unc119(+)]*
JAB197	*sep-1(erb-84[sep-1::linker::mScarlet])*
JAB212	*ify-1(erb-82[ify-1::linker::GFP])*
JAB221	*sep-1(erb-74[sep-1::linker::GFP]); ltIs37 [Ppie-1::mCherry::his-58 (pAA64); unc-119(+)] iv; unc-119(ed3) III*
JAB222	*ify-1(erb-82[ify-1::linker::GFP]); ltIs37 [Ppie-1::mCherry::his-58 (pAA64); unc-119(+)] iv; unc-119(ed3) III*

bioRxiv preprint doi: https://doi.org/10.1101/2023.12.12.571338; this version posted December 19, 2023. The copyright holder for this preprint (which was not certified by peer review) is the author/funder, who has granted bioRxiv a license to display the preprint in perpetuity. It is made available under aCC-BY 4.0 International license.

JAB248	*sep-1(erb-84[sep-1::linker::mScarlet]); ify-1(erb-82[ify-1::linker::GFP])*
JAB258	*sep-1(erb-74[sep-1::linker::GFP]); ltIs151 [pSO33; Pcpg-2::cpg-1SigSeq::mCherry-TEV-STag::cpg-2; unc-119(+)];ltIs37 [Ppie-1::mCherry::his-58 (pAA64); unc-119(+)] iv; unc-119(ed3)III*
JAB272	*sep-1(erb-84[sep-1::linker::mScarlet]);* ySi012 II; coh-4(tm1857), coh-3(gk112) V; *unc-119(ed3) III*
JAB275	*sep-1(erb-84[sep-1::linker::mScarlet]); pwIs281[Ppie-1:cav-1:GFP;unc-119(+)]; unc-119(ed3)III*
N2	*Bristol (wildtype)*
OCF1	*ojIs37[[Ppie-1::H2B::mCherry; unc-119(+)]]*
OD366	*unc-119(ed3) III; ltIs151 [pSO33; Pcpg-2::cpg-1SigSeq::mCherry-TEV-STag::cpg-2; unc-119(+)]*
RQ372	*unc-119(ed3)III, ojIs58[SEP-1::GFP; unc-119(+)]; ltIs37{pAA64(Ppie-1::mCherry::his-58); unc-119(+)]}IV*
TH214	*unc-119(ed3) III; ddIs128 [ify-1::TY1::EGFP::3xFLAG(92C12) + unc-119(+)].*
TY5430	*ySi012 II; coh-4(tm1857), coh-3(gk112) V; unc-119(ed3) III*
TY5431	*ySi012 II; ltIs37 [pAA64; Ppie-1::mCherry::his-58; unc-119(+)] IV; coh-4(tm1857), coh-3(gk112) V*
WH491	*unc-119(ed3) III, ojEx75[IFY-1::GFP unc119(+)]*

bioRxiv preprint doi: https://doi.org/10.1101/2023.12.12.571338; this version posted December 19, 2023. The copyright holder for this preprint (which was not certified by peer review) is the author/funder, who has granted bioRxiv a license to display the preprint in perpetuity. It is made available under aCC-BY 4.0 International license.

WH526	*unc-119(ed3) III, ojIs76[GFP::ify-1 unc119(+)]*

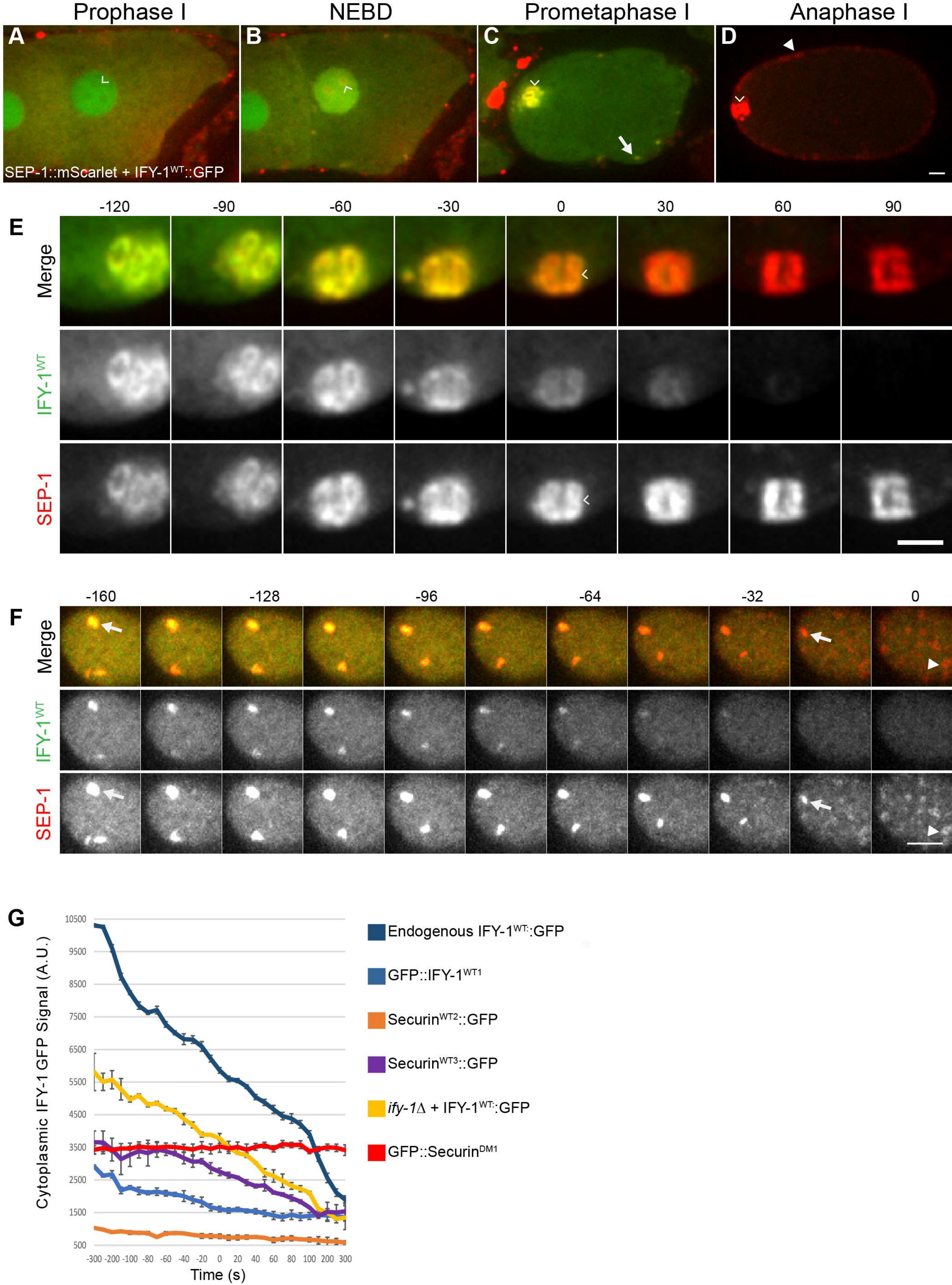

Prophase I
NEBD
Prometaphase I
Anaphase I
A
B
C
D
SEP-1::mScarlet + IFY-1WT::GFP
E
-120
-90
-60
-30
0
30
60
90
Merge
IFY-1WT
SEP-1
F
-160
-128
-96
-64
-32
0
Merge
IFY-1WT
SEP-1
G
Cytoplasmic IFY-1 GFP Signal (A.U.)
10500
9500
8500
7500
6500
5500
4500
3500
2500
1500
500
-300 -200 -100 -80 -60 -40 -20 0 20 40 60 80 100 200 300
Time (s)
Endogenous IFY-1WT::GFP
GFP::IFY-1WT1
SecurinWT2::GFP
SecurinWT3::GFP
ify-1Δ + IFY-1WT::GFP
GFP::SecurinDM1

A

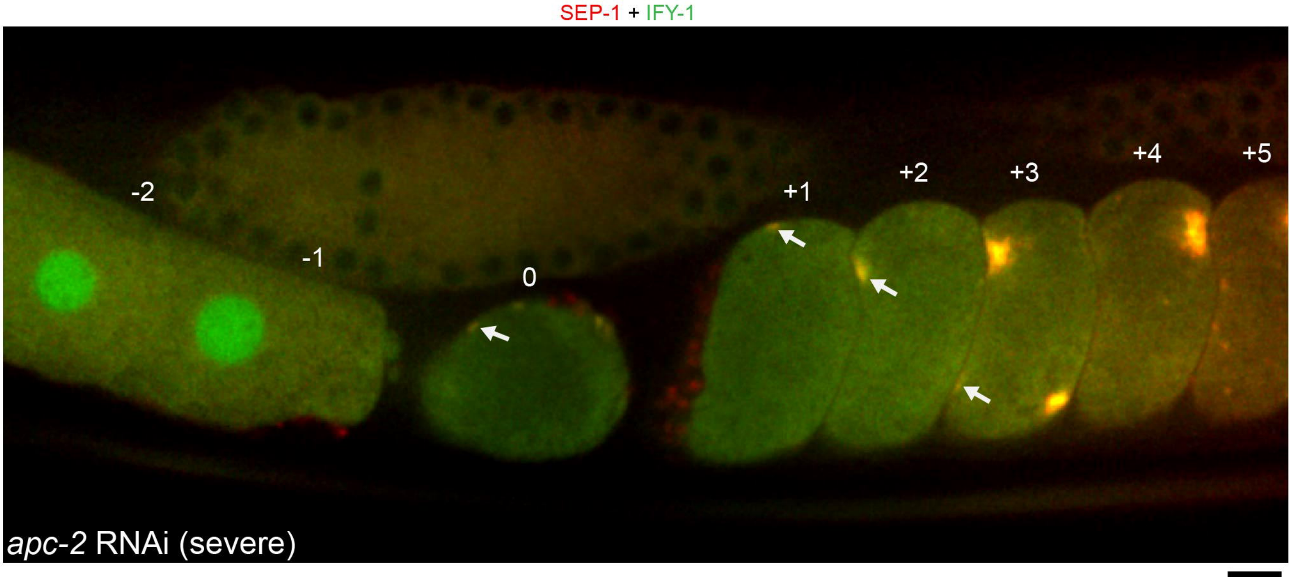

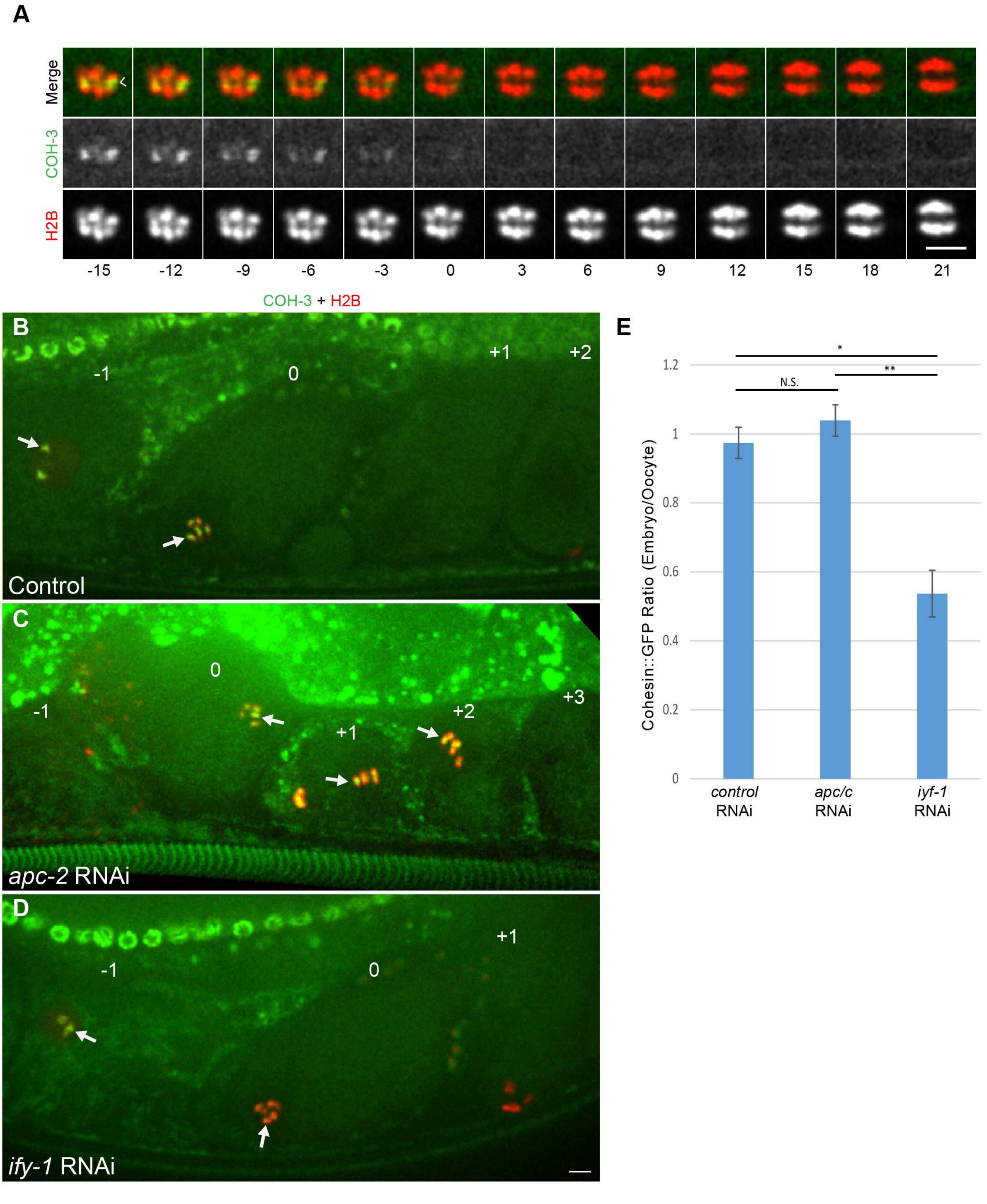
A
Merge
COH-3
H2B
-15
-12
-9
-6
-3
0
3
6
9
12
15
18
21
COH-3 + H2B
B
-1
0
+1
+2
Control
C
0
-1
+1
+2
+3
apc-2 RNAi
D
-1
0
+1
ify-1 RNAi
E
Cohesin::GFP Ratio (Embryo/Oocyte)
*
**
N.S.
0
0.2
0.4
0.6
0.8
1
1.2
control RNAi
apc/c RNAi
iyf-1 RNAi
